# A phase I dose escalation study of BIBW 2992, an irreversible dual inhibitor of epidermal growth factor receptor 1 (EGFR) and 2 (HER2) tyrosine kinase in a 2-week on, 2-week off schedule in patients with advanced solid tumours

**DOI:** 10.1038/sj.bjc.6604108

**Published:** 2007-11-20

**Authors:** F A L M Eskens, C H Mom, A S T Planting, J A Gietema, A Amelsberg, H Huisman, L van Doorn, H Burger, P Stopfer, J Verweij, E G E de Vries

**Affiliations:** 1 Department of Medical Oncology, Erasmus University Medical Center, Rotterdam, The Netherlands; 2 Department of Medical Oncology, University Hospital, Groningen, The Netherlands; 3 Boehringer Ingelheim Pharmaceuticals Inc, Ridgefield, CT, USA; 4 Boehringer Ingelheim BV, Alkmaar, The Netherlands; 5 Boehringer Ingelheim Pharma GmbH & Co. KG, Biberach, Germany

**Keywords:** phase I, BIBW 2992, epidermal growth factor receptor, tyrosine kinase inhibitor, pharmacokinetics

## Abstract

To assess tolerability, pharmacokinetics (PK), pharmacodynamics (PD) and clinical activity of the dual epidermal growth factor receptor (EGFR) 1 and 2 (HER2) tyrosine kinase inhibitor BIBW 2992. An escalating schedule of once-daily (OD) BIBW 2992 for 14 days followed by 14 days off medication was explored. Thirty-eight patients were enrolled. Dose levels were 10, 20, 30, 45, 70, 85, and 100 mg. At 100 mg dose-limiting toxicity (DLT) (common toxicity criteria grade 3 skin rash and grade 3 diarrhoea despite treatment with loperamide) occurred in two patients. In the next-lower dose of 70 mg, DLT (grade 3 fatigue and ALAT elevation) occurred in one of six patients. An intermediate dose level of 85 mg was studied. Here DLT occurred in two patients (grade 3 diarrhoea despite treatment and grade 2 diarrhoea lasting more than 7 days despite treatment). An additional 12 patients were treated at 70 mg. BIBW 2992 PK after single and multiple doses revealed moderately fast absorption, and no deviation from dose proportionality. Pharmacodynamics analysis in skin biopsies did not show significant changes in EGFR-associated biomarkers. However, a significant inhibitory effect on the proliferation index of epidermal keratinocytes was observed. No partial or complete responses were observed, stable disease lasting more than four cycles was seen in seven patients. The recommended dose for studies with BIBW 2992 for 14 days followed by 14 days off medication is 70 mg OD.

Increased expression of epidermal growth factor receptors (EGFR) is frequently found in epithelial tumours. EGFR and HER2 play a role in carcinogenesis, and alterations in these receptors are associated with poor outcome (Hynes and Lane, 2005). As HER2 overexpression is found to potentiate EGFR signalling, dual inhibition of these two receptors is considered to be of great potential clinical interest. A number of reversible and irreversible tyrosine kinase inhibitors have been explored in clinical trials, including the reversible dual inhibitor lapatinib, which has recently gained approval in the USA for patients with HER2-Neu overexpressing metastatic breast cancer progressing after trastuzumab (Baselga *et al*, 2002; Geyer *et al*, 2006).

BIBW 2992 (Figure 1) is an irreversible dual inhibitor of EGFR and HER2 tyrosine kinase. The inhibitory concentration (IC)_50_ is 0.5 nM for EGFR kinase and 14 nM for HER2 kinase. Related kinases from vascular endothelial growth factor 2, C-SRC and Lck are not inhibited at concentrations 100- to 1000-fold higher. BIBW 2992 inhibits EGF-induced EGFR phosphorylation and cellular proliferation in cell lines such as EGFR-overexpressing and HER2-expressing cell lines A431, NIH-3T3-HER2, NCI-N87 and BT-474 (Boehringer-Ingelheim, data on file). Oral BIBW 2992 induced tumour regression in mice carrying EGFR-overexpressing and HER2-expressing A431 xenografts, in mice carrying EGFR-overexpressing and HER2-expressing MDA-MB-453 xenografts, and in NCI-N87 gastric and SKOV-3 ovarian models at plasma concentrations of 80–280 nM (Boehringer-Ingelheim, data on file). Toxicity occurred in skin and kidneys in rats and the gastrointestinal tract in rats and minipigs.

Objectives of this study were (1) to determine maximum tolerated dose (MTD) and dose-limiting toxicity (DLT) when administered once-daily (OD) for 14 days followed by 14 days off treatment, (2) to characterise safety including acute and chronic adverse events (AE), (3) to characterise single- and multiple-dose pharmacokinetics (PK), (4) to analyse biomarkers in skin biopsies and (5) to assess antitumour activity.

## PATIENTS AND METHODS

### Eligibility criteria

Patients with histologically or cytologically confirmed solid tumours historically known to express EGFR and/or HER2, for whom no proven therapy exists or who are not amenable to established treatments were eligible. Additional eligibility criteria were as follows: age ⩾18 years; Eastern Cooperative Oncology Group (ECOG) performance status ⩽2; life expectancy ⩾3 months; adequate bone marrow (absolute neutrophil count ⩾1.5 × 10^9^ l^−1^, platelet count ⩾100 × 10^9^ l^−1^), hepatic (bilirubin ⩽26 *μ*mol l^−1^, serum alanine aminotransferase (ALT) and aspartate aminotransferase (AST) <3 times upper limit of normal (ULN) or <5 times ULN in case of liver metastases) and renal (creatinine ⩽132 *μ*mol l^−1^) function; no chemotherapy, immunotherapy, radiotherapy, or hormonal therapy within 28 days excluding LHRH agonists or hormones taken for breast cancer. A normal left ventricular ejection fraction (LVEF) based on echocardiography or MUGA scan was required. Specific exclusion criteria included impairment of gastrointestinal function interfering with drug absorption or chronic diarrhoea.

### Study design

BIBW 2992 was supplied by Boehringer-Ingelheim Pharma GmbH, Biberach, Germany as tablets of 5, 20, or 100 mg. Tablets were taken 1 h before breakfast. A cycle was defined as 14 days on treatment followed by 14 days off medication. At days of pharmacokinetic evaluation, BIBW 2992 was taken 1 h before breakfast. The starting dose corresponded with one-fifth of the toxic dose low in rats, the most sensitive species. The dose for successive cohorts was doubled until observation of drug-related AE grade ⩾2 according to common toxicity criteria v3.0 in one or more patients in the first cycle. Thereafter escalation steps were ⩽50%. Should DLT be observed in one out of six patients and dose escalation continued, escalation steps were ⩽35%. Initially, three patients were treated per dose level. If one patient experienced drug-related DLT, three additional patients were treated. When no further patient experienced DLT, the dose was escalated. When ⩾2/6 patients experienced DLT, three additional patients were treated 1 dose tier below unless six had already been treated there.

Maximum tolerated dose was defined as the highest dose with ⩽1/6 patients experiencing DLT during cycle 1. Dose-limiting toxicity was defined as grade 3 or 4 nonhaematological toxicity except untreated nausea, vomiting, or diarrhoea; grade 4 haematological toxicity; grade 3 diarrhoea, nausea, or vomiting despite supportive care; need for loperamide for ⩾7 days to treat or prevent grade ⩾2 diarrhoea; need for continuous administration of 5HT-3 antagonists or dexamethasone to treat or prevent grade ⩾2 nausea or vomiting; occurrence of grade ⩾2 cardiac left ventricular function or renal toxicity. In the absence of DLT or disease progression, patients received six cycles and were allowed to continue in an extension protocol. Intrapatient dose escalation was not allowed. The study was approved by local Ethics Committees, and all patients provided written informed consent.

### Pretreatment and follow-up studies

Prior to therapy, medical history taking and physical examination were performed. A complete blood cell count (CBC), white blood cell (WBC) differential and serum biochemistry were performed, as were urinalysis, electrocardiogram, MUGA scanning and, chest X-ray. Weekly evaluations during cycles 1 and 2 and every other week thereafter included physical examination, AE assessment, CBC, WBC differential and serum chemistry including renal function. Tumour measurements and MUGA scanning were performed every other cycle. Response was assessed using RECIST (Therasse *et al*, 2000).

### Pharmacokinetic sampling and data analysis

Blood samples (5 ml) were collected prior to dosing and 0.5, 1, 2, 3, 4, 5, 7, 9, and 24 h after drug administration on day 1 and 0.5, 1, 2, 3, 4, 5, 7, 9, 24, 48, and 72 h after drug administration on day 14 of cycle 1. Trough PK samples were taken before drug administration on day 8 of every cycle, whereas in every subsequent cycle, a sample was taken 24 h following day 14 dose. Samples were collected in pre-cooled EDTA-Vacutainer® tubes and centrifuged immediately. Plasma samples were stored at −20°C. Plasma concentrations of BIBW 2992 were analysed by validated high-performance liquid chromatography tandem mass spectrometry methods at Boehringer Ingelheim Pharma GmbH & Co. KG, Germany. The calibration curves for BIBW 2992 covered a range of 0.500–250 ng ml^−1^ plasma in undiluted samples.

Noncompartmental analysis was conducted using WinNonlin® (version 4.1, Pharsight, Mountainview, CA, USA). Standard noncompartmental methods were used to calculate area under the plasma concentration *versus* time curve (AUC_0−24,ss_), peak plasma concentration (*C*_max,ss_), apparent total body clearance (CL/*F*_ss_), apparent volume of distribution (*V*_z_/*F*_ss_), and terminal half-life (*t*_1/2,ss_). Accumulation ratio of *C*_max_ and AUC of days 1 and 14 were calculated (*R*_A,_ *C*_max_, and *R*_A,AUC_). Time to peak plasma concentration (*t*_max,ss_) was reported as median values.

### Pharmacodynamic determinations

Punch biopsies were taken from unaffected forearm skin before BIBW 2992 administration and at day 14 of cycle 1. Biopsies were fixed in 10% formalin at room temperature (RT) and embedded in paraffin. Tissue sections (4 *μ*m) were mounted on silan adhesive ‘Star Frost’ glass slides (Knittel Glaser, Braunschweig, Germany), dried overnight at 56°C, deparaffinised with xylene, and rehydrated. Expression of (1) Ki-67, (2) p27^KIP1^ (kinase inhibitory protein 1; KIP1), (3) EGFR, (4) phosphorylated mitogen-activated protein kinase (pMAPK), and (5) phosphorylated Akt (pAkt) was examined (Toyoshima and Hunter, 1994; Scholzen and Gerdes, 2000). Antibodies used were monoclonal mouse antihuman Ki-67 antigen (DakoCytomation, Glostrup, Denmark); monoclonal mouse antihuman p27 protein (Novacastra, Newcastle, UK); phospho-p44/42 Map kinase (Thr202/Tyr204) rabbit polyclonal antibody (Cell Signalling Technology, Beverly, MA, USA); phospho-Akt (Ser473) rabbit polyclonal antibody (Cell Signalling Technology), and monoclonal mouse anti-EGFR (clone E30; DakoCytomation). Isotype-matched negative control antibodies and negative control mouse IgG2a, kappa (DakoCytomation) were used to validate specificity of Ki-67, p27^KIP1^, and EGFR staining. Blocking peptides from SignalStain phospho-p44/42 (Thr 202/Tyr204) IHC detection kit (Cell Signalling Technology) and phospho-Akt (Ser473) blocking peptide (Cell Signalling Technology) were included to validate pMAPK and pAkt. Antibodies were diluted in antibody diluent (DakoCytomation) and all antigens but EGFR required heat-mediated (pressure cooker) antigen retrieval in sodium citrate (10 mM; pH 6.0). To unmask EGFR antigen, slides were incubated 3 min at RT in proteinase K solution. Endogenous peroxidase activity was blocked for 5 min at RT with peroxidase block and nonspecific cross-reactivity was blocked by incubation in 1% bovine serum albumin for 30 min at RT. Slides were incubated in diluted primary antibody and antigen detection by a two-step staining procedure using DAKO EnVision+ Polymer Detection System. Visualisation was achieved by incubation at RT for 3–5 min in 3,3′-diaminobenzidine chromogen-buffered substrate solution (DakoCytomation). Slides were counterstained with haematoxylin.

Ki-67- and p27^KIP1^-positive keratinocytes were scored by counting ⩾1000 cells. The number of pMAPK-, pAkt-, and EGFR-positive epidermal keratinocytes and staining intensity were estimated using the Allred scoring system (Toyoshima and Hunter, 1994). The proportion of positive cells was scored on a 0–5 scale and intensity on a 0–3 scale. Scoring of positive cells is based on epidermal keratinocytes.

### Statistical methods

Paired Student's *t*-tests were employed to calculate *P*-value for comparison of means and to define any statistically significant changes in biomarker levels. Correlations among initial biomarker levels were assessed by calculating Spearman's rank correlation coefficients.

## RESULTS

Thirty-eight patients (Table 1) received 114 cycles of BIBW 2992. Sequentially, dose levels studied were 10 mg (*n*=3), 20 mg (*n*=3), 30 mg (*n*=3), 45 mg (*n*=3), 70 mg (*n*=18), 100 mg (*n*=2), and 85 mg (*n*=6). Number of patients and cycles administered as a function of schedule and dose are listed in Table 2.

### Safety

Mild gastrointestinal AE, mainly diarrhoea and also nausea and vomiting, were observed in 35 patients at all dose levels. Diarrhoea was manageable with loperamide and usually was self-limiting, requiring no medication in the 14 drug-free days. Nausea and vomiting were mild and never required specific treatment. One patient (dose level, 70 mg) experienced reversible grade 2 stomatitis in cycle 1 and refused additional treatment.

Dose-dependent cutaneous AE manifested as dry skin, folliculitis, or skin rash and was frequently observed at all dose levels. Skin rash was located on face and trunk. In nine patients, minocycline 100 mg OD during the 14 treatment days alleviated or prevented symptoms. Skin toxicity was self-limiting and disappeared during the 14 drug-free days.

Other AE included mild fatigue in 24 patients and self-limiting epistaxis without changes in coagulation parameters in 20 patients. ALT, AST, alkaline phosphatase, and bilirubin elevations were seen at all dose levels. Grade 3 ALT (*n*=2), AST (*n*=1), and alkaline phosphatase (*n*=1) were observed in the 70 mg group. Grade 2 ALT was observed in three patients and four patients had grade 2 AST. Grade 2 alkaline phosphatase was seen in five and grade 2 bilirubin in two patients. Consistently, elevations of liver enzymes and bilirubin were related to the 14-day dosing schedule, with levels decreasing after drug discontinuation.

Haematological AE consisted of grade 1 anemia in 6 and grade 1 thrombocytopaenia in two patients.

Two patients showed LVEF reduction. In one patient at 10 mg, LVEF decreased from 76 to 56% after cycle 2 and to 35% after cycle 4 (grade 3). MUGA scan after cycle 4 was performed in another hospital. Two months after discontinuation of BIBW 2992, repeat assessment at the original hospital showed an LVEF of 54%. A transient decrease, despite normal ECGs and absence of clinical signs of heart failure, cannot be excluded. Left ventricular ejection fraction in a patient at 70 mg decreased from 56 to 40% after cycle 2 without signs of heart failure and with normal ECGs. No follow-up is available on this patient.

### Dose-limiting toxicity

At 70 mg, one DLT was observed in cycle 1, consisting of grade 3 ALT and fatigue.

**Figure 2:**
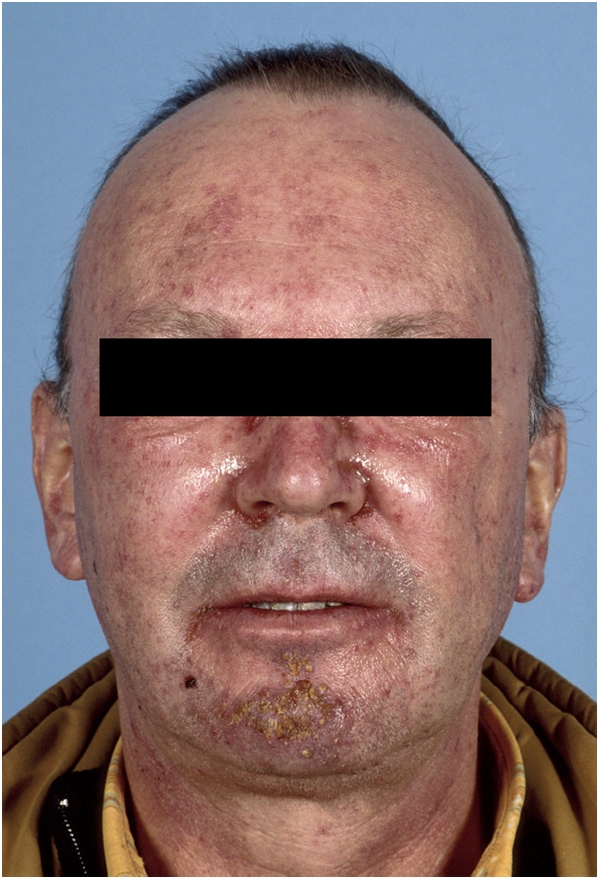
Grade 3 facial skin rash with ulceration and desquamation (BIBW 2992 100 mg OD).

At 100 mg, DLTs consisting of grade 3 skin rash (*n*=1) (Figure 2) and grade 3 diarrhoea despite loperamide (*n*=1) were observed in two patients. At 85 mg, DLTs were seen in two patients (grade 3 diarrhoea (*n*=1) and grade 2 diarrhoea lasting ⩾7 days despite loperamide (*n*=1)). Maximum tolerated dose was defined at 70 mg. At this dose, an additional 12 patients were studied of which two experienced DLT, consisting of grade 3 diarrhoea despite loperamide. Alltogether, at the recommended dose of 70 mg, 3 out of 18 patients experienced DLT.

### Pharmacokinetics

**Figure 3:**
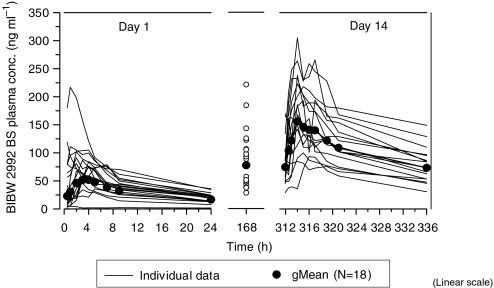
Individual (*n*=18/15) and gMean drug plasma concentration–time profiles of BIBW 2992 BS after multiple oral administration of 70 mg BIBW 2992 once-daily for 14 days to patients in treatment cycle 1 (linear scale).

Plasma concentration *versus* time curves of 70 mg BIBW 2992 on days 1 (single dose) and 14 (steady state) are shown in Figure 3. BIBW 2992 exhibited at least biexponential disposition kinetics. Similar disposition kinetics were observed for 10–100 mg on days 1 and 14.

Pharmacokinetic parameters of BIBW 2992 at steady state are displayed in Table 3; *t*_max,ss_ was 1–4 h postdose. Geometric mean (gMean) *C*_max,ss_ and exposure (AUC_0−24,ss_) increased over the tested dose range. There was no indication of a ‘nondose linear’ pharmacokinetic behaviour of BIBW 2992 with moderate-to-high inter-patient variability across the dose groups. Geometric mean *t*_1/2,ss_ ranged between 28 and 43 h. An apparent high total body clearance (CL/*F*_ss_) was determined, with gMean values 383–1390 ml min^−1^. BIBW 2992 exhibited high gMean *V*_z_/*F*_ss_ which might indicate extensive tissue distribution. Geometric mean values for *V*_z_/*F*_ss_ were 1250–4760 l. The apparent values obtained for total body clearance and volume of distribution should be treated with caution as the absolute bioavailability (*F*) of BIBW 2992 in humans is unknown. Steady state was reached at latest after 8 days of OD dosing. Geometric mean *R*_A,_ *C*_max_ was 1.76–2.74, and *R*_A,AUC_ was 2.42–3.82.

### Pharmacodynamics

**Figure 4:**
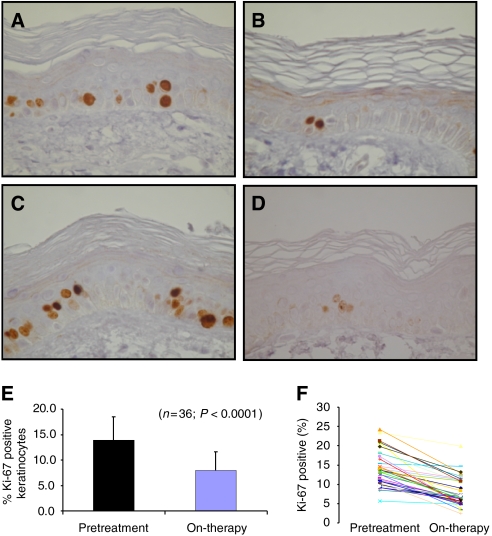
Ki-67 staining of paired skin biopsies of cancer patients treated with BIBW 2992. Pretreatment samples (**A** and **C**) and on-therapy sample treated with 10 mg day^−1^ (**B**) or at the MTD of 70 mg day^−1^. (**D**) Original magnification of the photomicrographs 400 × . Overall effect of BIBW 2992 treatment (all dose levels pooled) on percentage Ki-67-positive epidermal keratinocytes (mean±s.d.) in pretreatment *versus* on-therapy samples (**E**) and the effect of treatment for each individual patient (**F**).

In 36 paired skin biopsies, no consistent changes in EGFR-associated biomarkers (p27^KIP1^, EGFR, pMAPK, or pAkt) were detected. However, a marked reduction in epidermal keratinocytes proliferation index was observed (Figure 4A–E) with the percentage Ki-67-positive cells (means±s.d.) decreasing from 14.0±4.5 to 7.9±4.5% (*P*<0.0001). Only pMAPK expression and pAkt correlated (Pearson's *r*=0.356; *P*=0.045). Exploratory analyses found no relationship between pharmacodynamics markers and PK parameters.

### Antitumour activity

No partial or complete responses were observed. Eight patients, all treated at doses of ⩾45 mg, had minor tumour regressions. Stable disease lasting ⩾4 cycles was seen in seven patients with various tumour types, amongst which colorectal and nonsmall cell lung cancer. The median number of cycles in these patients was 6 (range 5–9). There was no relationship between occurrence of stable disease lasting for ⩾4 cycles and dose.

## DISCUSSION

This first-in-human study evaluated feasibility of oral administration of BIBW 2992 for 14 days followed by 14 days off treatment. Observed AE consisted of cutaneous toxicity, which was dose-limiting in individual patients, and a recognisable pattern of gastrointestinal AE, mainly consisting of diarrhoea, which was also dose-limiting. On the basis of these observations, the recommended phase 2 dose in this 14-day on, 14-day off schedule is 70 mg OD. Safety data obtained through observation of 12 additional patients treated at this dose confirmed the correctness of this conclusion.

In studies with EGFR tyrosine kinase inhibitors, cutaneous toxicity and diarrhoea are prominent side effects (Hidalgo *et al*, 2001; Baselga *et al*, 2002; Dittrich *et al*, 2002; Ranson *et al*, 2002; Hoekstra *et al*, 2005). Although these AE seem to have a common underlying mechanism, the exact pathogenesis is unknown. In this study rash, acne and dry skin showed an overall incidence of 95%. There was no clear relationship between dose of BIBW 2992 and frequency and/or type of skin event.

In this study, gastrointestinal AE showed a 92% incidence. These AE also occurred at all doses and consisted of diarrhoea, stomatitis, nausea, and vomiting. The incidence of stomatitis and epistaxis appeared to be dose-related. The single most frequently reported AE was diarrhoea, with an 84% incidence. In the first cycle, a relation between incidence of diarrhoea and stomatitis was observed. In addition, a relation between incidence of diarrhoea and BIBW 2992 dose was observed in the first cycle, and analysing all cycles, almost all patients developed episodes of diarrhoea. Except for three patients at the lower dose levels, diarrhoea started within 7 days. The onset of skin events was later than that of diarrhoea. It is known that the epithelium of the gastrointestinal tract contains EGFRs important for maintaining integrity of the mucosa and for mucosal repair. Inhibiting EGFRs could result in mucosal damage resulting in diarrhoea and/or mucositis (Playford *et al*, 1996). Other AE observed in this study were mild. Occurrences of asymptomatic increased liver enzymes and bilirubin were not clearly associated with liver metastases. Fluctuations followed the 14-day pattern of drug dosing and were quickly reversible after drug discontinuation. Hepatotoxicity, almost always reversible, is known to occur with another observed AEGFR and HER2 tyrosine kinase inhibitors (Geyer *et al*, 2006; Munster *et al*, 2007). In view of cardiac effects of trastuzumab, a decrease in cardiac function was considered to be a potential side effect of BIBW 2992. A change in LVEF was found in two patients in the absence of any cardiac symptoms. Preliminary data from ⩾120 patients from additional trials with BIBW 2992 have not indicated significant LVEF changes (Agus *et al*, 2006; Shaw *et al*, 2006). It remains unclear whether the observed changes were attributable to BIBW 2992 or occurred by chance.

Three other studies with BIBW 2992 with alternative schemes have been performed as part of a developmental programme (Agus *et al*, 2006; Lewis *et al*, 2006; Shaw *et al*, 2006). In two studies, a continuous dosing schedule was used, whereas a third study explored a 3-week on, 1-week off schedule. Preliminary results from all four studies support the concept that BIBW 2992 is well tolerated with gastrointestinal and skin toxicity being dose limiting in all studies. This pattern of toxicity indeed is completely in line with toxicities observed in phase 1 studies with, for example, both the reversible dual kinase inhibitor lapatinib and the irreversible selective EGFR inhibitor EKB-569 (Burris *et al*, 2005; Ehrlichman *et al*, 2006). Partial tumour responses have been observed in two patients with adenocarcinoma of the lung, with a complex heterozygous EGFR mutation in one patient. The patient has been on drug for ⩾14 months (Shaw *et al*, 2006). Additional studies of BIBW 2992 in nonsmall cell lung cancer are currently considered.

The PK profile revealed oral bioavailability, moderately fast absorption, and a *t*_1/2_ suitable for once-daily dosing. Maximum plasma concentrations and exposure increased with dose, and there appears to be dose linearity in the PK behaviour of BIBW 2992. All PK parameters displayed moderate-to-high variability within the expected range for orally administered EGFR tyrosine kinase inhibitors. At 70 mg, *C*_max_ was 25- to 700-fold higher than required to inhibit *in vitro* EGFR phosphorylation and cellular proliferation.

Somewhat to our surprise, no obvious BIBW 2992-related changes in EGFR-associated biomarkers were observed in this study. However, BIBW 2992 reduced the proliferation index of epidermal keratinocytes, although no clear dose–effect relationship was noted. Whether this somewhat disappointing result can be explained by methodology, or whether analysis of other signal pathways could have demonstrated biological activity of BIBW 2992 currently remains speculative. In contrast, and as mentioned earlier, hints of clinical activity have been observed.

Recently, lapatinib added to capecitabine in trastuzumab-refractory metastatic breast cancer patients has demonstrated clinical efficacy (Geyer *et al*, 2006). BIBW 2992 targets the same receptors as lapatinib but in an irreversible manner. This is a potential advantage, because receptor inhibition can only be overcome by newly synthesised EGFR and HER2. In a xenograft model, >24 h inhibition of EGFR phosphorylation was seen with the irreversible tyrosine kinase inhibitor EKB-569, even though the plasma half-life of this drug was 2 h (Torrance *et al*, 2000). Treatment with BIBW 2992 may therefore result in a sustained inhibition of EFGR and HER2. Furthermore, in preclinical studies the irreversible EGFR inhibitors EKB-569, CI-1033 (a pan-ErbB inhibitor), and HKI-272 (a dual EGFR/HER2 inhibitor) have shown inhibition of gefitinib-resistant kinases (Carter *et al*, 2005; Kwak *et al*, 2005). Acquired or primary resistance to gefitinib or erlotinib in NSCLC may therefore be circumvented by irreversible tyrosine kinase inhibitors. Thus, there are some potential advantages of irreversible tyrosine kinase inhibitors, such as BIBW 2992, over reversible inhibitors. However, their exact role remains to be established in future studies.

In summary, BIBW 2992 is a novel irreversible EGFR and HER2 tyrosine kinase inhibitor. When given daily for 2 weeks every 4 weeks, it is well tolerated, with AE being rash, diarrhoea, and transaminase elevations. These AE are reversible, and the pharmacokinetic profile is predictable and compatible with OD dosing.

## Figures and Tables

**Figure 1:**
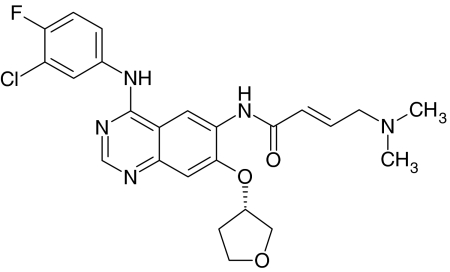
Chemical structure of BIBW 2992.

**Table 1:** Patient characteristics

Total	38
Male/female	19/19
Median age, years	58
Range, years	18–81
	
*Prior chemotherapy*	
None	7
1–2 prior regimens	20
⩾3 prior regimens	11
	
*Prior radiotherapy*	
No	23
Yes	15
	
*Prior hormonal therapy*	
No	36
Yes	2
	
Tumour type	
Colorectal carcinoma	10
Oesophageal carcinoma	5
Renal cell carcinoma	3
Nonsmall cell lung cancer	3
ACUP	3
Pancreatic carcinoma	2
Miscellaneous	12

**Table 2:** Dose escalation scheme, treatment duration

**Dose level (mg)**	**No. of patients**	**Total no. of cycles**	**No. of patients with DLT in cycle 1**
10	3	7	—
20	3	10	—
30	3	6	—
45	3	14^a^	—
70	18	48^b^	3
85	6	16^c^	2
100	2	7^d^	1

Abbreviation: DLT=dose-limiting toxicity.

Administration scheme: BIBW 2992 once-daily 2 weeks every 4 weeks.

a One patient with a parotic gland tumour received three additional cycles in the extension study.

b One patient with a gallbladder carcinoma received one additional cycle in the extension study.

c Two patients received one and five cycles respectively at 70 mg following the onset of DLT in the first treatment cycle.

d One patient received only one cycle that was complicated by DLT, one patient received cycles 3 and 4 at 85 mg due to DLT in the second cycle at 100 mg, and subsequently received cycles 5 and 6, as well as two additional cycles in the extension study at 70 mg.

**Table 3:** Geometric mean (and CV%) pharmacokinetic parameters of BIBW 2992 BS on day 14 after single oral administration of 10, 20, 30, 45, 70, 85, and 100 mg OD BIBW 2992 tablets (for *t*_max,ss_ median (and range) is displayed)

**Day 14**								
**BIBW 2992 dose**		**10 mg**	**20 mg**	**30 mg**	**45 mg**	**70 mg**	**85 mg**	**100 mg**
**No. of patients**		***n*=3**	***n*=3**	***n*=3**	***n*=3**	***n*=16**	***n*=4**	***n*=1**
*C*_max,ss_	(ng ml^−1^)	14.1 (72.1)	16.2 (51.9)	111 (69.7)	68.4 (31.7)	180 (34.5)	163 (53.0)	234
*t*_max,ss_	(h)	2.98 (2.00–4.00)	4.00 (4.00–4.00)	0.950 (0.517–2.07)	3.00 (2.00–4.07)	2.00 (0.500–5.00)	2.00 (2.00–2.02)	1.00
AUC_0−24,ss_	(ng h ml^−1^)	199 (56.9)	241 (59.4)	1310 (57.5)	840 (35.0)	2620 (36.3)	2340 (47.6)	2750
*t*_1/2, ss_	(h)	35.9 (12.0)	39.7 (13.4)	37.7 (25.1)	43.4 (31.4)	39.6 (38.7)	30.8 (12.9)	28.6
CL/*F*_ss_	(ml min^−1^)	839 (56.9)	1390 (59.4)	383 (57.5)	893 (35.0)	445 (36.3)	604 (47.6)	606
*V*_z_/*F*_ss_	(l)	2610 (42.8)	4760 (49.3)	1250 (64.0)	3350 (57.3)	1530 (33.5)	1610 (36.4)	1500
*R*_A,Cmax_		1.76 (34.0)	2.23 (7.62)	1.90 (23.9)	2.27 (13.7)	2.74 (111)	2.01 (65.6)	2.19
*R*_A, AUC_		2.42 (33.7)	2.61 (3.65)	2.62 (36.8)	2.45 (12.9)	3.82 (116)	2.50 (66.7)	2.86

Abbreviations: OD=once daily.
